# Impacts of Uniform Magnetic Field and Internal Heated Vertical Plate on Ferrofluid Free Convection and Entropy Generation in a Square Chamber

**DOI:** 10.3390/e23060709

**Published:** 2021-06-03

**Authors:** Chinnasamy Sivaraj, Vladimir E. Gubin, Aleksander S. Matveev, Mikhail A. Sheremet

**Affiliations:** 1 Department of Mathematics, PSG College of Arts & Science, Coimbatore 641014, Tamil Nadu, India; vcsivaraj@gmail.com; 2 School of Energy & Power Engineering, National Research Tomsk Polytechnic University, 634050 Tomsk, Russia; gubin@tpu.ru (V.E.G.); matveev@tpu.ru (A.S.M.); 3 Department of Theoretical Mechanics, Tomsk State University, 634050 Tomsk, Russia

**Keywords:** uniform Lorentz force, free convection, nanosuspension, non-uniformly warmed sheet, computational analysis

## Abstract

The heat transfer enhancement and fluid flow control in engineering systems can be achieved by addition of ferric oxide nanoparticles of small concentration under magnetic impact. To increase the technical system life cycle, the entropy generation minimization technique can be employed. The present research deals with numerical simulation of magnetohydrodynamic thermal convection and entropy production in a ferrofluid chamber under the impact of an internal vertical hot sheet. The formulated governing equations have been worked out by the in-house program based on the finite volume technique. Influence of the Hartmann number, Lorentz force tilted angle, nanoadditives concentration, dimensionless temperature difference, and non-uniform heating parameter on circulation structures, temperature patterns, and entropy production has been scrutinized. It has been revealed that a transition from the isothermal plate to the non-uniformly warmed sheet illustrates a rise of the average entropy generation rate, while the average Nusselt number can be decreased weakly. A diminution of the mean entropy production strength can be achieved by an optimal selection of the Lorentz force tilted angle.

## 1. Introduction

Optimization of technical systems in different engineering fields is related to both the energy transport intensification and liquid circulation control [1,2]. Today, an addition of nanoparticles of low concentration to a base fluid can increase the heat transfer rate [3,4], and as a result, there are many possible applications of nanofluids in various engineering fields including heat exchangers, solar collectors, building insulation, and others [4,5,6,7,8]. It is well known that control of flow structures can be achieved by using the magnetic field in the case of magneto-receptive medium [9]. The latter can be obtained by addition of ferric oxide nanoparticles to the host liquid. It is interesting to note that ferric oxide nanoparticles can be used for the drug delivery under the influence of a weak magnetic field [10]. The liquid systems with ferric oxide nanoparticles should be effective for the heat transfer augmentation and to exclude the possible technical system failure. Therefore, the entropy generation minimization theory can help to perform a theoretical analysis of such systems [11,12,13]. Some interesting and useful results regarding MHD natural convection and entropy generation in closed chambers have been already obtained [14,15,16,17,18,19,20,21,22,23,24]. Mahmoudi et al. [14] have numerically analyzed thermal convection and entropy production in a trapezoidal chamber filled with copper–water nanosuspension with isoflux local heater and isothermal vertical and inclined walls. Employing the single-phase nanosuspension approach with Patel, Brinkman, and Maxwell correlations for nanofluid heat conductivity, viscosity, and electrical conductivity, respectively, authors have studied an influence of the Rayleigh and Hartmann number and local heater position. It has been revealed that the average entropy production strength diminishes with nanoparticle concentration and rises with magnetic field intensity. The behavior of power-law non-Newtonian nanofluid and entropy production in a differentially warmed chamber under the horizontal magnetic field influence have been examined by Kefayati [15]. Using the lattice Boltzmann technique, an impact of Hartmann and Rayleigh numbers, nanoparticles volume fraction, and power-law index on nanofluid circulation and energy transport has been scrutinized. Obtained outcomes have demonstrated that all irreversibilities augment with nanoparticle concentration, while a reduction can be found with the growth of the power-law index. Kefayati and Tang [16] have calculated MHD thermogravitational energy transference and entropy production in a differentially heated chamber filled with power-law nanosuspension on the basis of a two-phase Buongiorno nanofluid model. Employing the lattice Boltzmann method in combination with the finite difference technique, authors have shown that a growth of the power-law index and Hartmann number can reduce the average entropy generation rate. Ghasemi and Siavashi [17] have performed a computational analysis of thermal convection and irreversibility nature in a porous chamber filled with copper–water nanofluid under an impact of isothermal vertical walls and horizontal uniform magnetic field. Analysis of Rayleigh and Hartmann numbers, thermal conductivity ratio, and nanoparticles concentration influence on entropy production has allowed the revelation that an instability impact can be found in entropy generation analysis. Thus, irreversibilities due to energy transference and liquid friction are the majority for *Ra* = 10^3^ and 10^5^, while for *Ra* = 10^4^, one can find a comparable influence of all mechanisms included in the analysis of irreversibility. Other useful results regarding entropy generation can be found in [18,19,20,21,22,23].

An influence of local isoflux heater and uniform Lorentz force on thermal convection and entropy production of a copper–water nanosuspension in an inclined porous chamber has been studied computationally by Rashad et al. [24]. Employing the finite difference technique, authors have demonstrated that average total entropy production reduces with nanoadditives’ volume fraction and rises with local heater size. Astanina et al. [25] have computed thermal convection and irreversibility analysis of ferroliquid in a semi-open trapezoidal chamber having a porous layer under the magnetic field influence. The obtained outcomes have shown that an inclusion of nanoadditives and Lorentz force strength reduce the average total entropy generation strength. Parveen and Mahapatra [26] have numerically scrutinized thermal and concentration convection with entropy production in a wavy chamber saturated with alumina-water nanosuspension under an influence of local isothermal heater and uniform Lorentz force. Employing the central differences and Bi-CGStab method for partial differential governing equations based on the single-phase nanofluid approach, authors have ascertained a reduction of average total entropy production with nanoparticles concentration and buoyancy ratio. Elshehabey et al. [27] have examined the thermogravitational energy transference and irreversibility nature of ferroliquid in an inclined semi-open wavy chamber under uniform magnetic field influence using non-linear Boussinesq approximation. Employing finite element method, researchers have demonstrated that the Boussinesq characteristic has a positive influence on the ferrosuspension circulation and major magnitudes of entropy production owing to liquid friction. Thermogravitational energy transference of non-Newtonian ferrofluid in a complex chamber under an impact of uniform Lorentz force has been investigated by Afsana et al. [28]. Authors have shown that a raise of the power-law index can augment the average total entropy production strength in the presence of Lorentz force, while the mean total entropy production can be reduced with the power-law index in the absence of Lorentz force.

At the same time, other interesting and useful questions for analysis of nanofluid behavior are about the effective thermal properties of nanofluids, and an influence of Brownian diffusion, thermophoresis, and interfacial layer on these properties is very useful [6,29,30,31,32].

This brief review shows that MHD thermal convection and entropy production in closed and semi-open chambers are very topical due to a wide variety of engineering problems where such formulation can be used. Moreover, the entropy generation minimization technique is very effective for analysis of technical systems where fluid circulation and energy transference theory can be used. The objective of this research is a numerical investigation of thermogravitational energy transport and irreversibility analysis in a closed cabinet saturated with a ferroliquid with a vertical hot sheet and uniform Lorentz force. It should be noted that this research is a continuation of analysis published by Sivaraj and Sheremet [33] for the horizontal heated plate. The horizontal heated plate has an essential influence on the circulation within the chamber due to large flow resistance [33], while for the vertical heated plate, this effect is weak, and more significant heat transfer enhancement can be found. Therefore, the innovation of the present study is a heat transfer enhancement in a closed chamber due to an influence of a heated inner vertical plate having non-uniform temperature and ferrofluid behavior under an influence of uniform magnetic field.

## 2. Mathematical Formulation

**Figure 1:**
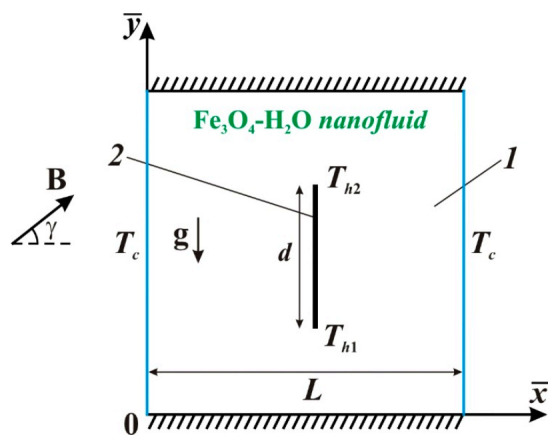
A sketch of the problem: *1*—nanofluid space; *2*—warmed vertical block.

The sketch for the considered thermal convection in a square ferrofluid chamber and the coordinates are demonstrated in Figure 1. The analyzed region is saturated with Fe_3_O_4_-H_2_O nanosuspension (*1* in Figure 1) and includes a non-uniformly warmed vertically oriented block (*2* in Figure 1). The temperature of this block depends linearly on the *y*-coordinate over the block and has a range between *T_h_*_1_ and *T_h_*_2_ (*T_h_*_1_ < *T_h_*_2_). The chamber horizontal borders are thermally insulated, and the vertical borders are isothermal at fixed temperature *T_c_* < *T_h_*_1_,*T_h_*_2_. Thermal attributes of the working liquid are constant, and the laminar motion mode is studied. The nanosuspension is Newtonian and heat-conducting; the Boussinesq approach is employed for description of the buoyancy force influence. The host liquid and the solid nanoadditives are in heat equilibrium. An inclined uniform magnetic field influences the convective liquid circulation and energy transport within the chamber.

Employing these approaches, the control equations are

(1) $$\frac{\partial \bar{u}}{\partial \bar{x}}+\frac{\partial \bar{v}}{\partial \bar{y}}=0$$

(2) $$\rho_{nf}\;\left(\frac{\partial \bar{u}}{\partial t}+\bar{u}\frac{\partial \bar{u}}{\partial \bar{x}}+\bar{v}\frac{\partial \bar{u}}{\partial \bar{y}}\right)\;=-\frac{\partial \bar{p}}{\partial \bar{x}}+\mu_{nf}\;\left(\frac{\partial^{2}\bar{u}}{\partial {\bar{x}}^{2}}+\frac{\partial^{2}\bar{u}}{\partial {\bar{y}}^{2}}\right)\;+\;\sigma_{nf}B^{2}\left[\bar{v}\cdot \cos\left(\gamma \right)-\bar{u}\cdot \sin\left(\gamma \right)\right]\sin\left(\gamma \right)$$

(3) $$\begin{array}{l} \rho_{nf}\;\left(\frac{\partial \bar{v}}{\partial t}+\bar{u}\frac{\partial \bar{v}}{\partial \bar{x}}+\bar{v}\frac{\partial \bar{v}}{\partial \bar{y}}\right)\;=-\frac{\partial \bar{p}}{\partial \bar{y}}+\mu_{nf}\;\left(\frac{\partial^{2}\bar{v}}{\partial {\bar{x}}^{2}}+\frac{\partial^{2}\bar{v}}{\partial {\bar{y}}^{2}}\right)\;+\;{\left(\rho \beta \right)}_{nf}g\left(T-T_{c}\right)\;+\\ +\sigma_{nf}B^{2}\left[\bar{u}\cdot \sin\left(\gamma \right)-\bar{v}\cdot \cos\left(\gamma \right)\right]\cos\left(\gamma \right) \end{array}$$

(4) $${\left(\rho c\right)}_{nf}\;\left(\frac{\partial T}{\partial t}+\bar{u}\frac{\partial T}{\partial \bar{x}}+\bar{v}\frac{\partial T}{\partial \bar{y}}\right)\;=k_{nf}\;\left(\frac{\partial^{2}T}{\partial {\bar{x}}^{2}}+\frac{\partial^{2}T}{\partial {\bar{y}}^{2}}\right)$$

With the following border restrictions

(5) $$\begin{array}{ll} t=0\;:\; & \bar{u}=\bar{v}=0,\;T=T_{c},\;\mathrm{at}\;0\leq \bar{x}\leq L\;\mathrm{and}\;0\leq \bar{y}\leq L,\\ t>0\;:\; & \bar{u}=\bar{v}=0,\;T=T_{c},\;\mathrm{at}\;\bar{x}=0,\;L\;\mathrm{and}\;0\leq \bar{y}\leq L,\\ & \bar{u}=\bar{v}=0,\;\frac{\partial T}{\partial \bar{y}}=0,\;\mathrm{at}\;\bar{y}=0,\;L\;\mathrm{and}\;0<\bar{x}<L,\\ & \bar{u}=\bar{v}=0,\;T=\bar{y}\frac{T_{h2}-T_{h1}}{d}+\frac{T_{h1}+T_{h2}}{2}-L\frac{T_{h2}-T_{h1}}{2d}\;\mathrm{at}\;\mathrm{the}\;\mathrm{vertical}\;\mathrm{block} \end{array}$$

Definition of nanofluid thermal properties can be found in [33].

The nanofluid heat conductivity is described by

(6) $$\frac{k_{nf}}{k_{f}}=\frac{k_{p}+2k_{f}-2\phi \left(k_{f}-k_{p}\right)}{k_{p}+2k_{f}+\phi \left(k_{f}-k_{p}\right)}$$

The nanofluid viscosity is

(7) $$\mu_{nf}=\frac{\mu_{f}}{{\left(1-\phi \right)}^{2.5}}$$

The nanofluid electrical conductivity is

(8) $$\sigma_{nf}=\sigma_{f}\left\{1+\frac{3\left(\sigma_{p}/\sigma_{f}-1\right)\phi }{\left(\sigma_{p}/\sigma_{f}+2\right)-\left(\sigma_{p}/\sigma_{f}-1\right)\phi }\right\}$$

Employing the non-dimensional parameters

(9) $$\begin{array}{l} x=\frac{\bar{x}}{L},\;y=\frac{\bar{y}}{L},\;u=\frac{\bar{u}L}{\alpha_{f}},\;v=\frac{\bar{v}L}{\alpha_{f}},\;p=\frac{\bar{p}L^{2}}{\rho_{nf}\alpha_{f}^{2}},\\ \tau =\frac{t\alpha_{f}}{L^{2}},\;\theta =\frac{T-T_{c}}{\Delta T},\;\Delta T=\frac{T_{h1}+T_{h2}}{2}-T_{c} \end{array}$$

The non-dimensional control equations are

(10) $$\frac{\partial u}{\partial x}+\frac{\partial v}{\partial y}=0$$

(11) $$\begin{array}{l} \frac{\partial u}{\partial \tau }+u\frac{\partial u}{\partial x}+v\frac{\partial u}{\partial y}=-\;\frac{\partial p}{\partial x}+\left(\frac{\mu_{nf}\;\rho_{f}}{\mu_{f}\;\rho_{nf}}\right)\;Pr\;\left(\frac{\partial^{2}u}{\partial x^{2}}+\frac{\partial^{2}u}{\partial y^{2}}\right)+\\ +\left(\frac{\sigma_{nf}\;\rho_{f}}{\sigma_{f}\;\rho_{nf}}\right)\;Ha^{2}Pr\;\left[v\;\cos\left(\gamma \right)-u\;\sin\left(\gamma \right)\right]\;\sin\left(\gamma \right) \end{array}$$

(12) $$\begin{array}{l} \frac{\partial v}{\partial \tau }+u\frac{\partial v}{\partial x}+v\frac{\partial v}{\partial y}=-\;\frac{\partial p}{\partial y}+\left(\frac{\mu_{nf}\;\rho_{f}}{\mu_{f}\;\rho_{nf}}\right)Pr\;\left(\frac{\partial^{2}v}{\partial x^{2}}+\frac{\partial^{2}v}{\partial y^{2}}\right)+\left(\frac{\beta_{nf}}{\beta_{f}}\right)\;Ra\;\;Pr\;\theta +\\ +\left(\frac{\sigma_{nf}\;\rho_{f}}{\sigma_{f}\;\rho_{nf}}\right)\;Ha^{2}Pr\;\left[u\;\sin\left(\gamma \right)-v\;\cos\left(\gamma \right)\right]\;\cos\left(\gamma \right) \end{array}$$

(13) $$\frac{\partial \theta }{\partial \tau }+u\frac{\partial \theta }{\partial x}+v\frac{\partial \theta }{\partial y}=\;\left(\frac{\alpha_{nf}}{\alpha_{f}}\right)\;\;\left(\frac{\partial^{2}\theta }{\partial x^{2}}+\frac{\partial^{2}\theta }{\partial y^{2}}\right)$$

With the following boundary restrictions

(14) $$\begin{array}{ll} \tau =0\;:\; & u=v=0,\;\theta =0,\;\mathrm{at}\;0\leq x\leq 1\;\mathrm{and}\;0\leq y\leq 1,\\ \tau >0\;:\; & u=v=0,\;\theta =0,\;\mathrm{at}\;x=0,\;1\;\mathrm{and}\;0\leq y\leq 1,\\ & u=v=0,\;\frac{\partial \theta }{\partial y}=0,\;\mathrm{at}\;y=0,\;1\;\mathrm{and}\;0<x<1,\\ & u=v=0,\;\theta =1+\frac{\lambda }{\delta }\left(2y-1\right)\;\mathrm{at}\;\mathrm{vertical}\;\mathrm{block} \end{array}$$

The employed non-dimensional characteristics are

(15) $$Pr=\frac{\nu_{f}}{\alpha_{f}},\;Ra=\frac{g\beta_{f}\Delta T\;L^{3}}{\nu_{f}\alpha_{f}},\;Ha=BL\sqrt{\frac{\sigma_{f}}{\mu_{f}}},\;\delta =\frac{d}{L},\;\lambda =\frac{T_{h2}-T_{h1}}{2\;\Delta T}$$

The local energy transport intensity over the vertical walls is

(16) $$Nu=-\;\left(\frac{k_{nf}}{k_{f}}\right)\;\frac{\partial \theta }{\partial x}$$

As a result, the mean Nusselt number is

(17) $$\bar{Nu}=\int_{0}^{1}\left(Nu_{\mathrm{left}\;\;\mathrm{wall}}+Nu_{\mathrm{right}\;\;\mathrm{wall}}\right)\;\;dy$$

An appearance of irreversibility sources in the temperature and velocity fields including fluid friction and energy transference characterizes the irreversibility behavior. The dimensional local entropy production *s_gen_* is

(18) $$\begin{array}{l} s_{gen}=\frac{k_{nf}}{T^{2}}\left[{\left(\frac{\partial T}{\partial \bar{x}}\right)}^{2}+{\left(\frac{\partial T}{\partial \bar{y}}\right)}^{2}\right]+\frac{\mu_{nf}}{T}\left[2\;{\left(\frac{\partial \bar{u}}{\partial \bar{x}}\right)}^{2}+2\;{\left(\frac{\partial \bar{v}}{\partial \bar{y}}\right)}^{2}+{\left(\frac{\partial \bar{u}}{\partial \bar{y}}+\frac{\partial \bar{v}}{\partial \bar{x}}\right)}^{2}\right]+\\ +B^{2}\;\frac{\sigma_{nf}}{T}{\left[\bar{u}\;\sin\left(\gamma \right)-\bar{v}\;\cos\left(\gamma \right)\right]}^{2} \end{array}$$

Here, each term characterizes the local entropy production owing to heat transfer $\left(s_{gen,ht}\right)$, fluid friction $\left(s_{gen,ff}\right)$, and outer magnetic field $\left(s_{gen,mf}\right)$.

The non-dimensional local entropy generation $S_{gen}$ is

(19) $$S_{gen}=s_{gen}\;\frac{T^{2}L^{2}}{k_{f}\;\Delta T^{2}}=S_{gen,ht}+S_{gen,ff}+S_{gen,mf}$$

where

(20) $$S_{gen,ht}=\frac{k_{nf}}{k_{f}}\;\frac{1}{\Omega^{2}\;{\left(\theta +\Omega^{-1}\right)}^{2}}\;\;\left[{\left(\frac{\partial \theta }{\partial x}\right)}^{2}+{\left(\frac{\partial \theta }{\partial y}\right)}^{2}\right]$$

(21) $$S_{gen,ff}=\frac{\mu_{nf}}{\mu_{f}}\;\frac{1}{\Omega^{2}\;\left(\theta +\Omega^{-1}\right)}\frac{Ge}{Ra}\;\;\left[2\;{\left(\frac{\partial u}{\partial x}\right)}^{2}+2\;{\left(\frac{\partial v}{\partial y}\right)}^{2}+{\left(\frac{\partial u}{\partial y}+\frac{\partial v}{\partial x}\right)}^{2}\right]$$

(22) $$S_{gen,mf}=\frac{\sigma_{nf}}{\sigma_{f}}\;\frac{1}{\Omega^{2}\;\left(\theta +\Omega^{-1}\right)}\frac{Ge}{Ra}\;{{}^{Ha}}^{2}\;{\left[u\;\sin\left(\gamma \right)-v\;\cos\left(\gamma \right)\right]}^{2}$$

These Equations (20)–(22) include the dimensionless thermal difference (Ω) and the Gebhart number (*Ge*)

(23) $$\Omega =\frac{\Delta T}{T_{c}}\;\mathrm{and}\;Ge=\frac{g\beta_{f}L}{c_{f}}$$

The dimensionless mean entropy generation is

(24) $$S_{gen,avg}=\frac{1}{\vartheta }\int S_{gen}d\vartheta =S_{gen,ht,avg}+S_{gen,ff,avg}+S_{gen,mf,avg}$$

The Bejan number *Be* is found to be

(25) $$Be=\frac{S_{gen,ht}}{S_{gen,ht}+S_{gen,ff}+S_{gen,mf}}$$

The average Bejan number *Be_avg_* is

(26) $$Be_{avg}=\frac{S_{gen,ht,avg}}{S_{gen,ht,avg}+S_{gen,ff,avg}+S_{gen,mf,avg}}$$

## 3. Numerical Procedure

The control Equations (10)–(13) with restrictions (14) have been approximated using the finite volume technique and a uniform staggered mesh with the SIMPLE procedure of Patankar [34]. The QUICK model of Hayase et al. [35] and central differences have been applied for convective and diffusive members of equations. For the border restrictions, a third order scheme has been also used [35]. The obtained system of equations has been solved by Thomas procedure. The steady state outcomes have been achieved using the following convergence criteria

(27) $$\frac{\sum_{i,\;j}\left|\;\xi_{i,j}^{m}-\xi_{i,j}^{m-1}\;\right|}{\sum_{i,\;j}\left|\;\xi_{i,j}^{m}\;\right|}\leq 10^{-7}$$

Here, *ξ* characterizes *u*, *v*, or *θ*; the parameter *m* characterizes the iteration number and (*i*,*j*) are for the Cartesian coordinates.

The developed technique has been validated for different test problems [35]. Thus, for the thermal nanosuspension convection in a chamber, the results of Ghasemi et al. [36] have been used. Table 1 demonstrates a good agreement for the mean *Nu* at *Ra* = 10^5^, *Ha* = 0, and various *φ*.

The in-house computational program has been validated for the free convection and irreversibility analysis in a chamber with a hot block [37]. Table 2 shows a good agreement for the total entropy production rate at *Ra* = 10^5^, *δ* = 0.5, and different Ω in comparison with [37].

The mesh independence test has been performed using four grids of 103 × 103, 203 × 203, 303 × 303, and 403 × 403 nodes. Table 3 demonstrates an influence of mesh parameters on ${\left|\psi \right|}_{\max}$ for *Ra* = 10^7^, *Ha* = 50, *γ* = 0°, *φ* = 0.04, and *λ* = 0. As a result, a mesh of 303 × 303 has been chosen for the further analysis.

## 4. Results and Discussion

**Figure 2:**
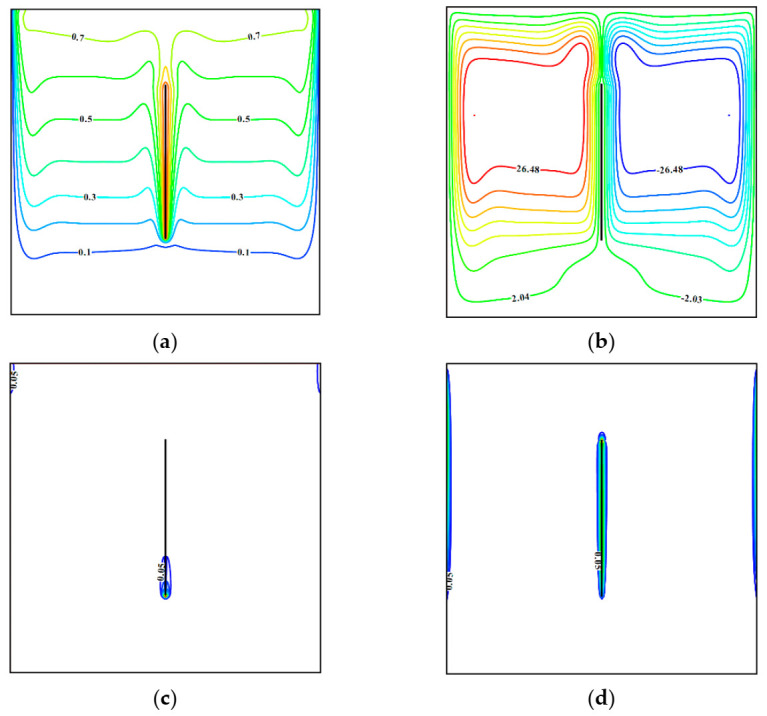
Isolines of (**a**) *θ*, (**b**) *ψ*, (**c**) *S_gen_*_,*ht*_, (**d**) *S_gen_*_,*ff*_ for Ω = 0.01, *φ* = 0.04, *λ* = 0, *Ha* = 0.

**Figure 3:**
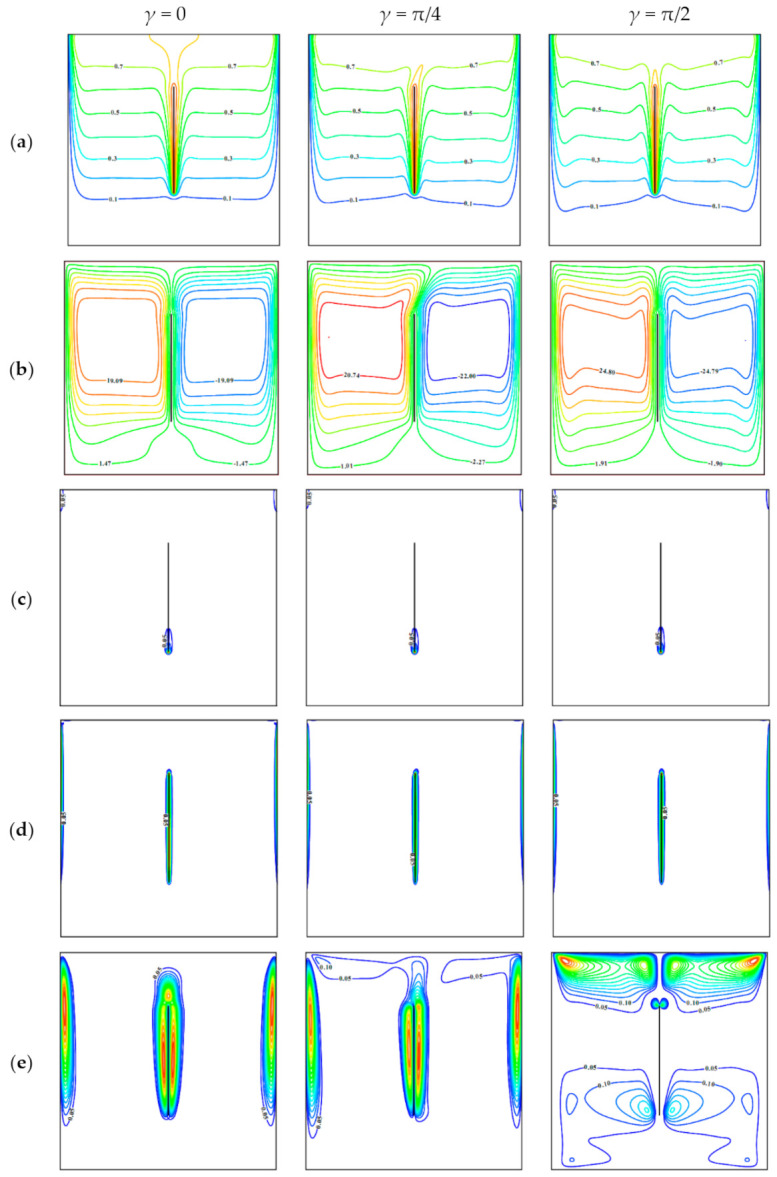
(**a**) Isotherms, (**b**) streamlines, local entropy production owing to (**c**) energy transport, (**d**) nanosuspension friction, and (**e**) Lorentz force for Ω = 0.01, *φ* = 0.04, *λ* = 0, *Ha* = 50, and different *γ*.

**Figure 4:**
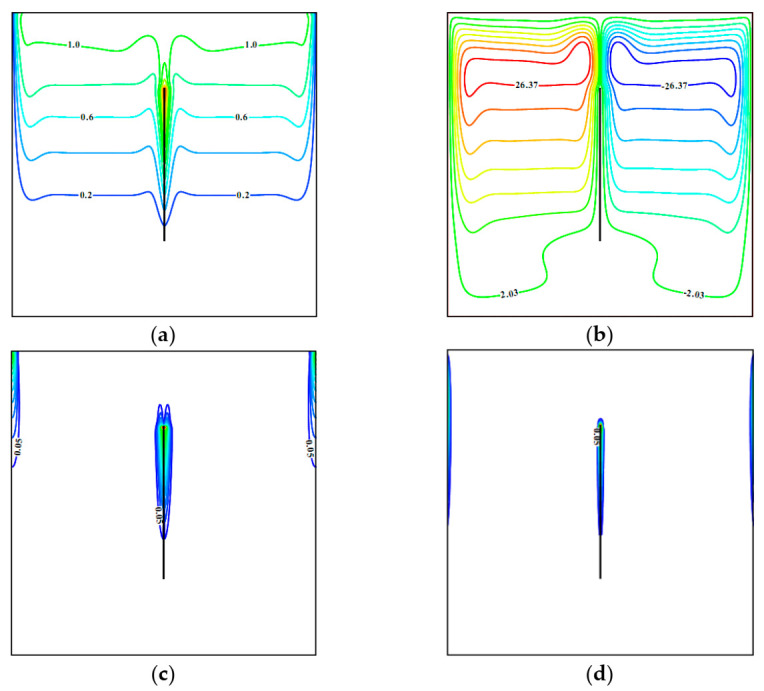
Isolines of (**a**) *θ*, (**b**) *ψ*, (**c**) *S_gen_*_,*ht*_, (**d**) *S_gen_*_,*ff*_ for Ω = 0.01, *φ* = 0.04, *λ* = 1, *Ha* = 0.

**Figure 5:**
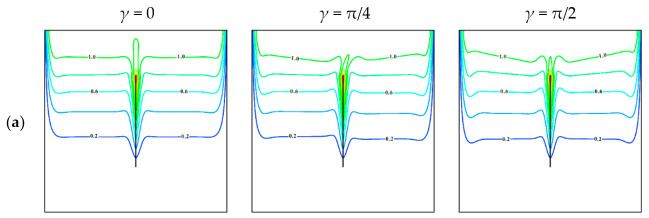

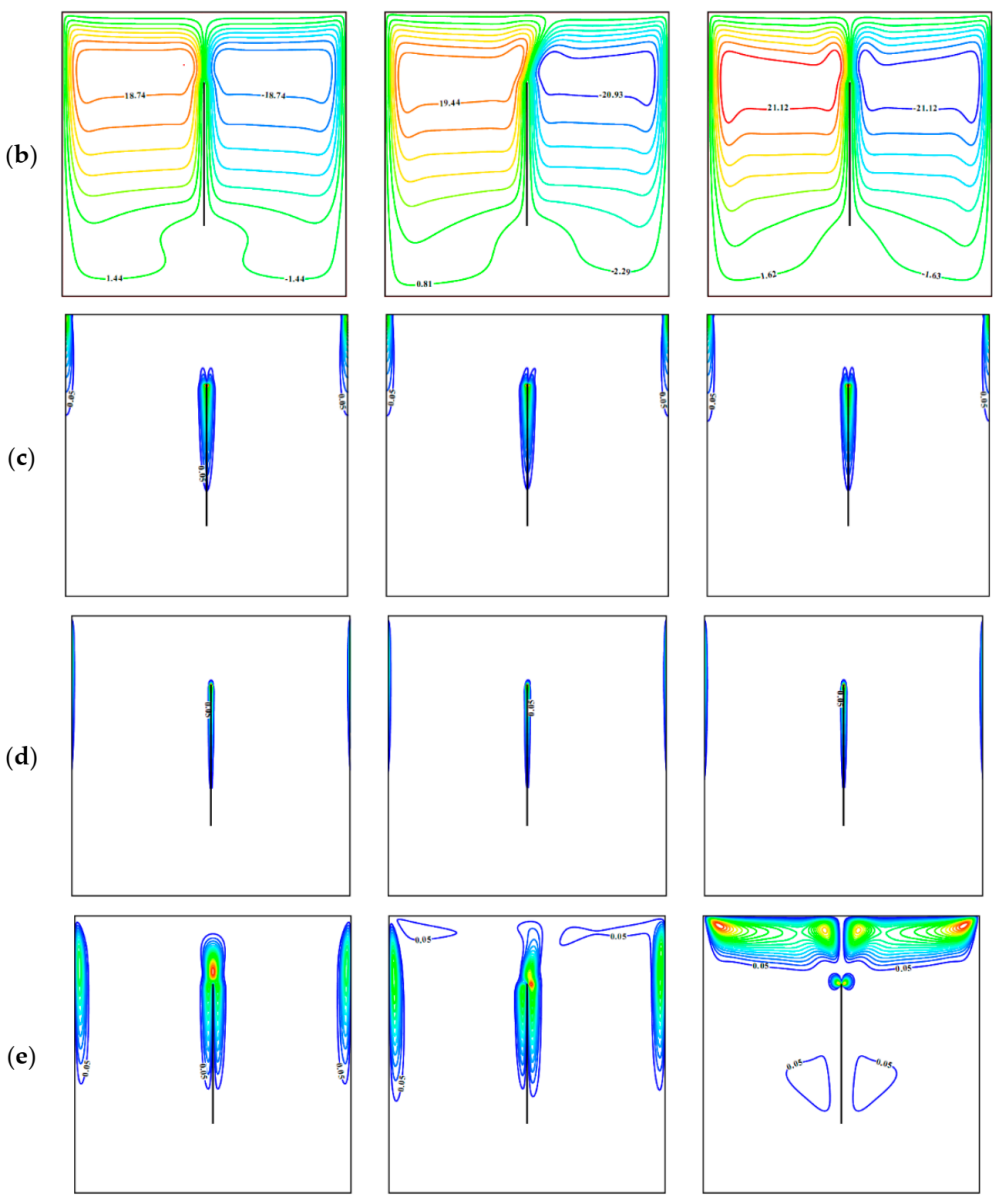
Isolines of (**a**) *θ*, (**b**) *ψ*, (**c**) *S_gen_*_,*ht*_, (**d**) *S_gen_*_,*ff*_, (**e**) *S_gen_*_,*mf*_ for Ω = 0.01, *φ* = 0.04, *λ* = 1, *Ha* = 50, and different *γ*.

**Figure 6:**
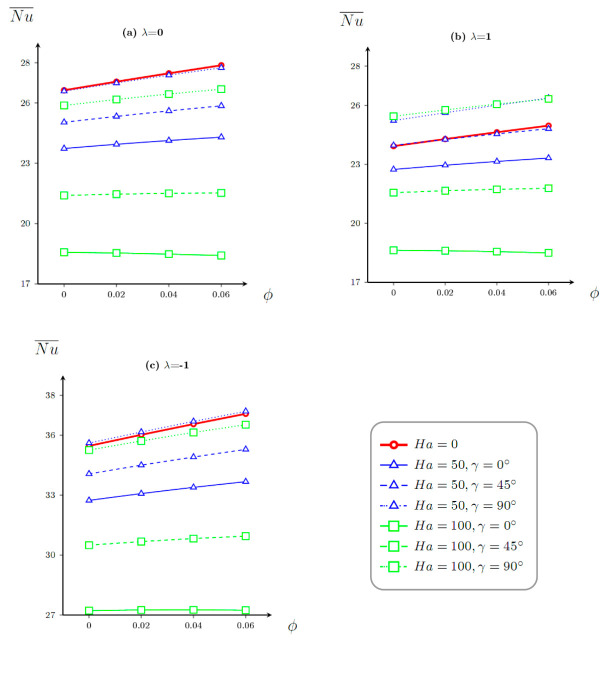
Dependences of mean *Nu* on *φ* for (**a**) *λ* = 0, (**b**) *λ* = 1, and (**c**) *λ* = –1.

**Figure 7:**
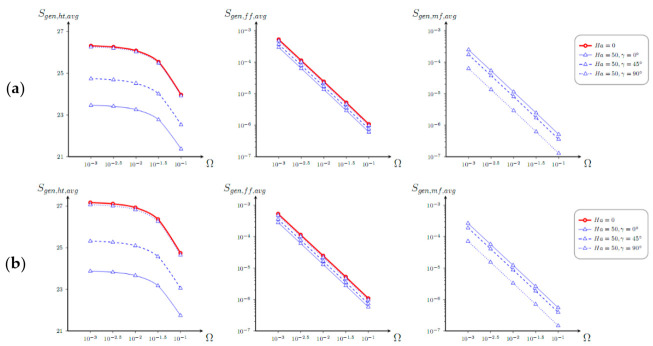
Dependence of mean entropy production caused by the energy transference, liquid friction, and Lorentz force with Ω for *λ* = 0, (**a**) *φ* = 0, and (**b**) *φ* = 0.04 and various *Ha* and *γ*.

**Figure 8:**
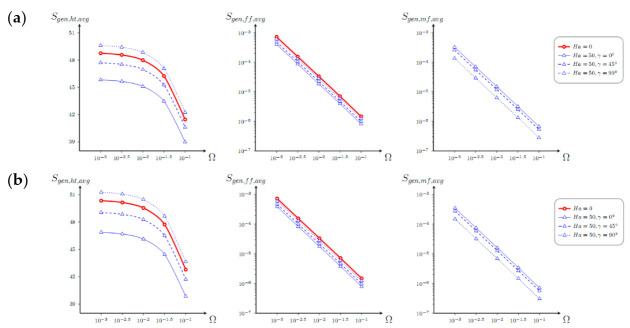
Dependence of mean entropy production caused by the energy transference, liquid friction, and Lorentz force with Ω for *λ* = 1, (**a**) *φ* = 0, and (**b**) *φ* = 0.04 and various *Ha* and *γ*.

An impact of Lorentz force with various inclination angles on free convection and irreversibility behavior inside a nanoliquid-filled cabinet with a centered hot vertical plate has been scrutinized. The effects of the nanoparticles’ concentration (*φ* = 0.0 and 0.04), plate non-uniformity characteristic (*λ* = –1, 0, 1), Hartmann number (*Ha* = 0–100), magnetic field inclination angle (*γ* = 0°–π/2°), and non-dimensional temperature drop (Ω = 0.001–0.1) on nanosuspension motion and energy transference are scrutinized for *Pr* = 6.8377, *δ* = 0.5, and *Ra* = 10^7^. The calculated outcomes are demonstrated employing isotherms, streamlines, and local entropy production as well as mean Nusselt number, mean total entropy production strength, and mean Bejan number in Figure 2, Figure 3, Figure 4, Figure 5, Figure 6, Figure 7 and Figure 8.

Figure 2 demonstrates isolines for Ω = 0.01, *φ* = 0.04, *λ* = 0, and *Ha* = 0. It should be noted that *λ* = 0 characterizes a uniform heating of the plate, namely, the plate is an isothermal vertical block. Isothermal lines illustrate a formation of thermally stratified cores on the right and left sides from the plate where warming of the space occurs from the top zone to the lower one due to the heat conduction. Such temperature distribution can be added by the streamlines formed within the chamber, where two convective cells reflect an appearance of upstream flows close to the centered heated plate and two descending flows near the side cooled borders. Local entropy production isolines caused by the energy transference illustrate an appearance of a significant temperature drop in the bottom part of the sheet where the heated body interacts with cold nanofluid. At the same time, an influence of nanosuspension friction occurs within the velocity boundary layers formed on the isothermal vertical surfaces. Therefore, centered plate and upper parts of vertical cavity borders are characterized by high-velocity gradients. It is worth noting that a presence of side borders’ upper parts in this distribution is explained by an interaction of more intensive flows in the top part of these walls compared to the bottom ones.

An impact of the inclined Lorentz force on the mentioned local parameters is shown in Figure 3 for Ω = 0.01, *φ* = 0.04, *λ* = 0, and *Ha* = 50. An addition of Lorentz force suppresses the convective motion and energy transport within the chamber, taking into account Figure 2 and Figure 3. For *γ* = 0, when the magnetic field has the horizontal impact on the chamber, one can find more smooth isotherms without the local extreme near-vertical borders that characterizes less intensive circulation of the nanosuspension. Moreover, a shape of streamlines reflects uniform weak circulation of the liquid. Features of local entropy production patterns caused by the energy transference and circulation friction have been described above, and for Lorentz force influence, the behavior is repeated. As for the Lorentz force influence on the local entropy production, it should be highlighted that for *γ* = 0 zones of high entropy production caused by the magnetic influence are similar to the zones of high entropy production due to flow friction. For *γ* = π/4, one can find a symmetry loss for all considered functions; intensity of convective flow rises, but heating strength of the upper chamber part is decreased. It is useful to note that an inclusion of the Lorentz force with *γ* = π/4 reflects a distortion of heat plume above the centered sheet, and as a result, a border between two ascending flows over the plate is distorted to the right side, but an intensity of convection flow rises compared to the case of *γ* = 0. A symmetry loss in this case also can be found for the isolines of local entropy production due to the Lorentz force. In the case of *γ* = π/2 more intensive motion can be found, while this strength is less compared to the case of *Ha* = 0. More essential influence of Lorentz force in this case occurs in the upper part. It should be noted that intensification of the convective flow can be explained by collinearity of gravity force and magnetic field strength vector.

Figure 4 demonstrates isolines of stream function, temperature, and local entropy production caused by the energy transference and flow friction for Ω = 0.01, *φ* = 0.04, *Ha* = 0, and the non-uniformly heated plate, when temperature if the plate rises from the bottom end until the upper one. In this case, more essential heating of the chamber occurs, but strength of the convective flow is decreased slightly due to not so essential temperature gradient near this centered plate. At the same time, cores of convective cells are located in the upper part, and zones of more intensive entropy production owing to the energy transference and circulation friction are along the upper parts of vertical surfaces.

An addition of magnetic field for non-uniformly warmed vertical sheet reflects a degradation of convective flow and energy transport (see Figure 5). It should be noted that all changes in analyzed local parameters are similar to those mentioned in Figure 3, but for the base presented in Figure 4. It means that distortion and variation of all isolines with magnetic influence of *Ha* = 50 have been discussed in the case of uniform heating, but for the non-uniform heating, all changes are superimposed on the patterns presented in Figure 4.

Influence of magnetic field, nanoparticles’ volume fraction, and heating of the centered plate direction on the mean *Nu* is shown in Figure 6. In the case of uniform heating of the vertical plate, the heat transfer enhancement with nanoparticles’ concentration can be found for low and moderate Hartmann number, while for high *Ha* (*Ha* = 100) with *γ* > π/4, the energy transference degradation with *φ* can be revealed. In this case, an inclusion of magnetic influence allows the diminishment of the energy transference strength. In the case of non-uniform warming of the plate with a change of temperature from small magnitudes in the bottom zone until high magnitudes in the upper zone (*λ* = 1), one can find less intensive energy transport and a similar impact of the Lorentz force inclination angle, as in the previous case (*λ* = 0), but in this case, an addition of magnetic field can intensify the heat transfer for moderate and high Hartmann numbers when magnetic field intensity vector is parallel to the gravity force. When non-uniform heating of the vertical plate occurs from the upper part to the bottom one, more intensive energy transference can be revealed compared to other mentioned cases.

Behavior of average entropy generation with Ω, *φ*, *Ha*, and *γ* for *λ* = 0 is shown in Figure 7. A growth of the non-dimensional temperature drop characterizes a diminution of the mean entropy production strength owing to each source. Moreover, irreversibility caused by the flow friction and Lorentz force is more sensitive to the influence of Ω, whilst the mean entropy production caused by the energy transference is not as sensitive. A rise of the nanoadditives’ concentration illustrates an increment of *S_gen_*_,*ht*,*avg*_, while *S_gen_*_,*ff*,*avg*_ and *S_gen_*_,*mf*,*avg*_ are not as sensitive to *φ*. An addition of magnetic field reduces the mean entropy production strength, but this diminution can be controlled by selection of the magnetic field orientation.

In the case of non-uniform heating of the vertical plate shown in Figure 8, a rise of *S_gen_*_,*ht*,*avg*_ can be achieved with a rise of magnetic field intensity, while nature of other parameters is similar to the investigated above for *λ* = 0.

## 5. Conclusions

MHD thermogravitational energy transference and irreversibility analysis in a square ferroliquid chamber under the influence of a centered vertical sheet has been studied numerically using the finite volume technique. An innovation of this research deals with possible heat transfer enhancement and entropy production minimization in a closed chamber with a vertical heated plate having non-uniform temperature under an impact of ferrofluid and uniform Lorentz force.

Impacts of different control parameters including Hartmann number, Lorentz force inclination angle, non-dimensional temperature drop, plate non-uniformity heating parameter on flow structures, and energy transport intensity have been discussed. The performed analysis has shown that an addition of uniform Lorentz force can intensify the convective energy transference in the case of non-uniform heating of the vertical plate, while for horizontal plate such situation is absent. Moreover, nanoparticles addition allows the diminishment of the heat transference rate for high Hartmann numbers and horizontal orientation of magnetic field, whilst for the horizontal internal sheet, an introduction of nanoparticles enhances the heat transference. For the vertical sheet, more essential energy transference augmentation can be found owing to a lack of the significant resistance from this plate location. Therefore, internal plate orientation and magnetic field intensity and orientation can be considered as a good control parameters for the energy transport within considered engineering systems. Taking into account these outcomes, the further investigation can be related to the influence of the heat-generating inner plate on the heat transfer patterns within the chamber and possible intensification of energy transport in combination with hybrid nanofluids impact.

## Figures and Tables

**Table 1:** Mean *Nu* for various *φ* compared to [36] for *Ra* = 10^5^, *Ha* = 0.

	*φ* = 0	*φ* = 0.02	*φ* = 0.04	*φ* = 0.06
Data [36]	4.738	4.820	4.896	4.968
Obtained outcomes	4.7306	4.8133	4.8908	4.9633

**Table 2:** Total entropy production rate for different Ω in comparison with [37].

Ω	0.02	0.04	0.06	0.08	0.1
Data [37]	6.701	6.576	6.457	6.342	6.231
Obtained outcomes	6.8539	6.7219	6.5952	6.4733	6.3558

**Table 3:** Mesh sensitivity analysis.

Grid	103 × 103	203 × 203	303 × 303	403 × 403
${\left|\psi \right|}_{\max}$	26.0023	25.8046	25.7620	25.7431

## Data Availability

All data is contained within this article.

## Abbreviations

The following abbreviations are used in this manuscript:

*B*	magnitude of the uniform magnetic field (kg⋅s^−2^⋅A^−1^)
*Be*	local Bejan number (–)
*Be_avg_*	average Bejan number (–)
*c*	heat capacity (J⋅kg^−1^⋅K^−1^)
*d*	dimensional length of the plate (m)
*g*	gravitational acceleration (m⋅s^−2^)
*Ge*	Gebhart number (–)
*Ha*	Hartmann number (–)
*k*	thermal conductivity (W⋅m^−1^⋅K^−1^)
*L*	size of the cavity (m)
*Nu*	local Nusselt number (–)
$\bar{Nu}$	average Nusselt number (–)
*p*	dimensionless pressure (–)
$\bar{p}$	dimensional pressure (Pa)
*Pr*	Prandtl number (–)
*Ra*	Rayleigh number (–)
$s_{gen}$	dimensional local entropy generation (W⋅m^−3^⋅K^−1^)
$S_{gen}$	dimensionless local entropy generation (–)
$S_{gen,avg}$	dimensionless average total entropy generation (–)
$s_{gen,ff}$	dimensional local entropy generation due to fluid friction (W⋅m^−3^⋅K^−1^)
$S_{gen,ff}$	dimensionless local entropy generation due to fluid friction (–)
$S_{gen,ff,avg}$	dimensionless average entropy generation due to fluid friction (–)
$s_{gen,ht}$	dimensional local entropy generation due to heat transfer (W⋅m^−3^⋅K^−1^)
$S_{gen,ht}$	dimensionless local entropy generation due to heat transfer (–)
$S_{gen,ht,avg}$	dimensionless average entropy generation due to heat transfer (–)
$s_{gen,mf}$	dimensional local entropy generation due to external magnetic field (W⋅m^−3^⋅K^−1^)
$S_{gen,mf}$	dimensionless local entropy generation due to external magnetic field (–)
$S_{gen,mf,avg}$	dimensionless average entropy generation due to external magnetic field (–)
*T*	dimensional temperature (K)
*t*	dimensional time (s)
*T_h1_, T_h2_*	dimensional temperature range at heated plate (K)
*T_c_*	dimensional temperature at cooled vertical walls (K)
$\bar{u},\bar{v}$	dimensional velocity components along horizontal and vertical directions (m⋅s^−1^)
*u, v*	dimensionless velocity components along horizontal and vertical directions (–)
$\bar{x}$	dimensional Cartesian coordinate measured along the bottom wall of the cavity (m)
$\bar{y}$	dimensional Cartesian coordinate measured along the vertical wall of the cavity (m)
*x, y*	dimensionless Cartesian coordinates (–)
*Greek symbols*	
*α*	thermal diffusivity (m^2^⋅s^−1^)
*β*	coefficient of thermal expansion (K^−1^)
*γ*	inclination angle of the magnetic field (–)
*δ*	dimensionless length of the plate (–)
*θ*	dimensionless temperature (–)
*λ*	temperature parameter (–)
*μ*	dynamic viscosity (Pa⋅s)
*ρ*	fluid density (kg⋅m^−3^)
*σ*	electrical conductivity of the fluid (Ω^−1^⋅m^−1^)
*τ*	dimensionless time (–)
*φ*	nanoparticles volume fraction (–)
*ψ*	dimensionless stream function (–)
**Ω**	dimensionless temperature difference (–)
*Subscripts*	
*c*	cold
*f*	fluid
*h*	hot
*nf*	nanofluid
*p*	particle
